# Effect of mixing on enzymatic hydrolysis of steam-pretreated spruce: a quantitative analysis of conversion and power consumption

**DOI:** 10.1186/1754-6834-4-10

**Published:** 2011-05-11

**Authors:** Benny Palmqvist, Magnus Wiman, Gunnar Lidén

**Affiliations:** 1 Department of Chemical Engineering, Lund University, Box 124, Se-221 00 Lund, Sweden

## Abstract

**Background:**

When scaling up lignocellulose-based ethanol production, the desire to increase the final ethanol titer after fermentation can introduce problems. A high concentration of water-insoluble solids (WIS) is needed in the enzymatic hydrolysis step, resulting in increased viscosity, which can cause mass and heat transfer problems because of poor mixing of the material. In the present study, the effects of mixing on the enzymatic hydrolysis of steam-pretreated spruce were investigated using a stirred tank reactor operated with different impeller speeds and enzyme loadings. In addition, the results were related to the power input needed to operate the impeller at different speeds, taking into account the changes in rheology throughout the process.

**Results:**

A marked difference in hydrolysis rate at different impeller speeds was found. For example, the conversion was twice as high after 48 hours at 500 rpm compared with 25 rpm. This difference remained throughout the 96 hours of hydrolysis. Substantial amounts of energy were required to achieve only minor increases in conversion during the later stages of the process.

**Conclusions:**

Impeller speed strongly affected both the hydrolysis rate of the pretreated spruce and needed power input. Similar conversions could be obtained at different energy input by altering the mixing (that is, energy input), enzyme load and residence time, an important issue to consider when designing large-scale plants.

## Introduction

When scaling up lignocellulose-based ethanol production, the desire to increase the final ethanol titer after the fermentation (a factor that strongly affects process economy) can introduce a number of problems. To achieve a high ethanol titer after fermentation, an increased content of water-insoluble solids (WIS) is needed in the enzymatic hydrolysis (EH) step; however, increasing the WIS content has been shown to have negative effects on EH, resulting in decreased yields [1]. Furthermore, high WIS content will result in high viscosities, which in turn will lead to high energy demands for efficient mixing. For instance, the viscosity of pretreated spruce at 12% WIS was recently found to be about 2 Pa s (at a shear rate (γ) of 50/s)[2].

Therefore, determining to what extent mixing is required to obtain an efficient EH is a key issue. Mais *et al. *[3] and Ingesson *et al. *[4] investigated the effects of different shaking regimes on the EH of cellulose. They both concluded that intermittent shaking (that is, mainly low-speed shaking but with shorter periods of intense shaking) was comparable with constant intense shaking. However, these experiments were conducted in 300 mL shake flasks with relatively low WIS content, and therefore do not represent an intensified process. Jorgensen *et al. *[5] and Roche *et al. *[6] both studied high WIS content (up to between 20% and 40%) in reactor systems consisting of rotating bottles/barrels, and concluded that efficient initial mixing is important to distribute the enzymes, but once the enzymes are distributed, rotation speed becomes less important. By contrast, studies using stirred bioreactors indicated a positive correlation between impeller speed and the rate of EH or performance in simultaneous saccharification and fermentation (SSF) [7-9]. However, these studies compared only two different impeller speeds and/or different impeller types to change the mixing in the reactor. To our knowledge, there have not been any systematic studies reporting the influence of mixing on the EH of pretreated lignocellulose, at realistic consistencies, and its effect on energy input for agitation.

In a stirred tank, the power consumption (P) is a function of impeller speed (N_i_) and diameter (D_i_), fluid density (ρ) and the impeller power number (P_0_), based on the equation:

P_0 _is a function of the Reynolds impeller number (Re_i _= ρ × N_i _× D_i_^2^/μ) and hence the fluid viscosity (μ), and also depends on the type of impeller and reactor. In turbulent regimes (Re_i _> 10^4^) however, the power number is constant, and only depends on the impeller and reactor design, but when operating in the laminar and transition region (Re_i_< 10^4^), the power dissipated by the impeller depends on the fluid viscosity. Because P_0 _depends on the materials viscosity (when Re_i_< 10^4^), rheological information about pretreated lignocellulosic materials is vital to make adequate calculations and designs for large-scale processes. Recently, a number of studies have focused on the rheological characterization of pretreated lignocellulosic materials, mainly corn stover [10-12], but also other materials such as red-oak sawdust [13] and spruce [2]. It has been shown that these pretreated materials, behave similar to shear-thinning fluids, and exhibit a yield stress (τ_γ_). Both viscosity and yield stress have been shown to increase rapidly with increasing WIS content, which complicates processes that strive for higher WIS content, because stagnant zones in the EH and/or SSF reactors are likely to occur. At laboratory scale, this is often dealt with by increasing the impeller speed to a point where complete motion of the fluid is achieved. At a larger scale, however, this would dramatically increase the power consumption needed to operate the impeller, a factor which would probably influence the process economy.

Studies on the required energy input for mixing during EH and/or SSF processes are, to our knowledge, surprisingly few. In technoeconomical calculations, the National Renewable Energy Laboratory (NREL) allowed about 60 W/m^3 ^for agitation in one of their SSF process designs [14]. This figure is a model-based estimation of power consumption for large-scale reactors (3,600 m^3^), but the value is low in comparison with experimental values obtained by Zhang *et al. *[15] for an SSF process employing both a helical ribbon impeller and a Rushton impeller in a 5 litre reactor. The latter authors reported power consumptions ranging from 9% up to as much as 59% of the heating value of the produced ethanol, values that are obviously far too high for an industrial process to be economically feasible.

In this work, we aimed to quantitatively investigate the effects of mixing on the rate of EH of steam-pretreated spruce. Pretreated material was enzymatically hydrolyzed in stirred reactors at different controlled stirring rates. Estimates of power consumption were made based on the rheological characteristics of the material, taking into account the change in rheology during the process. The results showed a strong effect of mixing on the rate of EH, and emphasize the interplay between mixing (that is, energy input), enzyme load and residence time as a means to reach a desired degree of substrate conversion.

## Methods

### Raw material and pretreatment

Chips (2 to 10 mm) from debarked Norway spruce (*Picea abies*) were provided by Witskövfle Sågverk AB, Sweden. The chips were impregnated with 2.5% SO_2 _(based on moisture content) for 20 minutes in plastic bags. The material was then steam-pretreated for 5 minutes at 210°C in a 10 litre reactor, as previously described by Palmqvist *et al. *[16]. The pretreated material was stored at 4°C. The composition of the solid and liquid fractions of the pretreated slurry (Table 1) were analyzed using NREL standard procedures [17,18], and the WIS content of the preatreated material was measured to be 13% (wt/wt) by washing the fibers repeatedly with deionized water over filter paper (number 1; Whatman, Florham Park, NJ, USA).

**Table 1:** Composition of solid and liquid fraction of the pretreated spruce

Solids	% of WIS^a^	Liquid	g/L
Glucan	48.0	Glucose	29.8
Mannan	1.9	Mannose	30.3
Galactan	0.8	Galactose	5.1
Xylan	0.5	Xylose	9.9
Lignin	45.0		

The WIS content was measured to 13%.

^a^Water-insoluble solids

### Hydrolysis experiments

All hydrolysis experiments were carried out in 2.5 litre bioreactors (Biostat A (B. Braun Biotech International, Melsungen, Germany) and Biostat A Plus (Sartorius, Melsungen, Germany)), with a diameter (D_t_) of 130 mm. The reactors were equipped with a pitched-blade impeller (three blades at an angle of 45°, a diameter (D_i_) of 70 mm and a blade width (w_i_) of 20 mm) pumping upwards. The working weight was 1.0 kg, corresponding to a volume of 980 mL and the liquid level reached 80 mm from the bottom. The whole pretreatment slurry was diluted with sterile, deionized water to a WIS content of 10%, which was the initial WIS concentration in the hydrolysis experiments. To ensure an even temperature distribution (34°C) even at low impeller speeds, at which stagnant zones developed, the reactors were placed in temperature-controlled water baths. The pH of the diluted slurry was set initially to 5.0 by addition of 10 mol/l NaOH. To prevent microbial contamination, 0.4 g/L of sodium azide was added. The enzyme preparation (Cellic CTec) had a cellulase activity of 95 filter-paper units (FPU)/g enzyme solution and a β-glucosidase activity of 590 IU/g enzyme solution (Novozymes A/S, Bagsvaerd, Denmark). Experiments were conducted at two different enzyme loadings, 10 and 20 FPU/g glucan, corresponding to a β-glucosidase activity of 62.1 and 124.2 IU/g glucan, respectively. The experiments with high enzyme loading were carried out in duplicate and excellent reproducibility was found, with the SD of the concentrations being < 4.2% for all samples. Each experiment was initiated by a short period (10 seconds) of intense mixing at 500 rpm to ensure an even initial distribution of the enzymes. This procedure was also carried out before every sampling to assure a representative sample. Samples for high-performance liquid chromatography (HPLC) analysis were taken repeatedly throughout the hydrolysis.

### Analysis

Determination of cellulase activity was performed according to standard NREL procedures [19]. The β-glucosidase activity was determined according to a method previously described by Berghem and Pettersson [20], which was slightly modified.

HPLC was used for analysis of sugars. Samples from the hydrolysis liquid were separated in a centrifuge (Z 160 M, Hermle Labortechnik, Wehingen, Germany) in 2 mL eppendorf tubes at 14,000 rpm for 5 minutes. The supernatant was filtered through 0.2 μm filters and stored at -20°C. The sugar concentrations, mainly glucose, were determined using a polymer column (Aminex HPX-87P; Bio-Rad Laboratories, München, Germany) at 85°C. Deionized water (Elga Maxima, Elga, Marlow, UK) was used as eluent, with a flow rate of 0.6 mL/min. The sugars were detected with a refractive index detector (Waters 2410, Waters, Milford, MA, USA).

### Power consumption estimations

Estimations of the power input for agitation at different impeller speeds were made by electrical measurements with a power meter. The energy dissipated to the liquid was approximated to the difference in motor-power requirement between 'agitating' air (which was regarded as the power drawn by the motor itself because of losses due to, for example, friction) and mixing the pretreatment slurry at 10% WIS content.

### Calculations

The dissipated power (P) at different impeller speeds (that is, at different Re_i_) was used to calculate the Power number,

A rheological characterization of the material used has previously been reported by Wiman *et al. *[2]. The pretreated material is non-newtonian, that is, viscosity changes with shear rate, and hence, impeller speed. The shear rate in the reactor is approximated to the average shear rate (γ_avg _= K_s _× N_i_) according to Metzner and Otto [21] for shear-thinning fluids, where N_i _is the impeller speed and K_s _is a weak function of impeller type. Because the exact value of K_s _was not known for the pitched-blade impeller, it was assigned a value of 11.5 in accordance with reports for Rushton turbines [21]. When calculating the viscosity () of the material at different impeller speeds and conversions it was assumed that K_PL _is a function of the WIS content only (that is, K_PL _= a × WIS^b^, according to Wiman *et al. *[2]) and hence of the degree of conversion during hydrolysis. The parameter n_PL _was considered to be constant throughout the hydrolysis. The yield stress of the material is rather low at the initial WIS concentration and then rapidly decreases during the hydrolysis [2]. Therefore, the power law model () was chosen to represent the viscosity in the reactor during the whole hydrolysis of 96 hours. Once the viscosity is estimated, the Reynolds impeller number can be calculated. The estimated power numbers and the corresponding Reynolds numbers were fitted using the least-squares method to an equation (P_0 _= K_1_/Re_i _+ K_2_) previously suggested by Wassmer and Hungenberg for non-newtonian fluids [22].

A response-surface statistical model, with conversion as a function of total energy input and hydrolysis time, was created in Matlab7.9.0 (Mathworks, Natick, USA) for the different enzyme loadings using a second-order polynomial to allow presentation of the results using a three-dimensional plot.

## Results

The aim of the study was to quantitatively investigate the effect of mixing on the EH of pretreated spruce, and to relate this effect to the needed energy input. Five different impeller speeds, ranging from 25 to 500 rpm, were chosen to represent different mixing regions, ranging from a low degree of mixing (where stagnant zones developed) to full mixing, at which complete motion of the whole slurry was achieved. The impeller speed needed to achieve complete fluid motion was initially (at 10% WIS) as high as 300 to 400 rpm, but decreased during the course of hydrolysis. Power measurements and rheological information about the material was used to estimate the power and energy input for the hydrolysis process, taking into account the changes in rheology throughout the process. The effect of enzyme loading was also studied to compare the capability of different process options in reaching a high conversion.

### Effect of impeller speed on the EH

**Figure 1:**
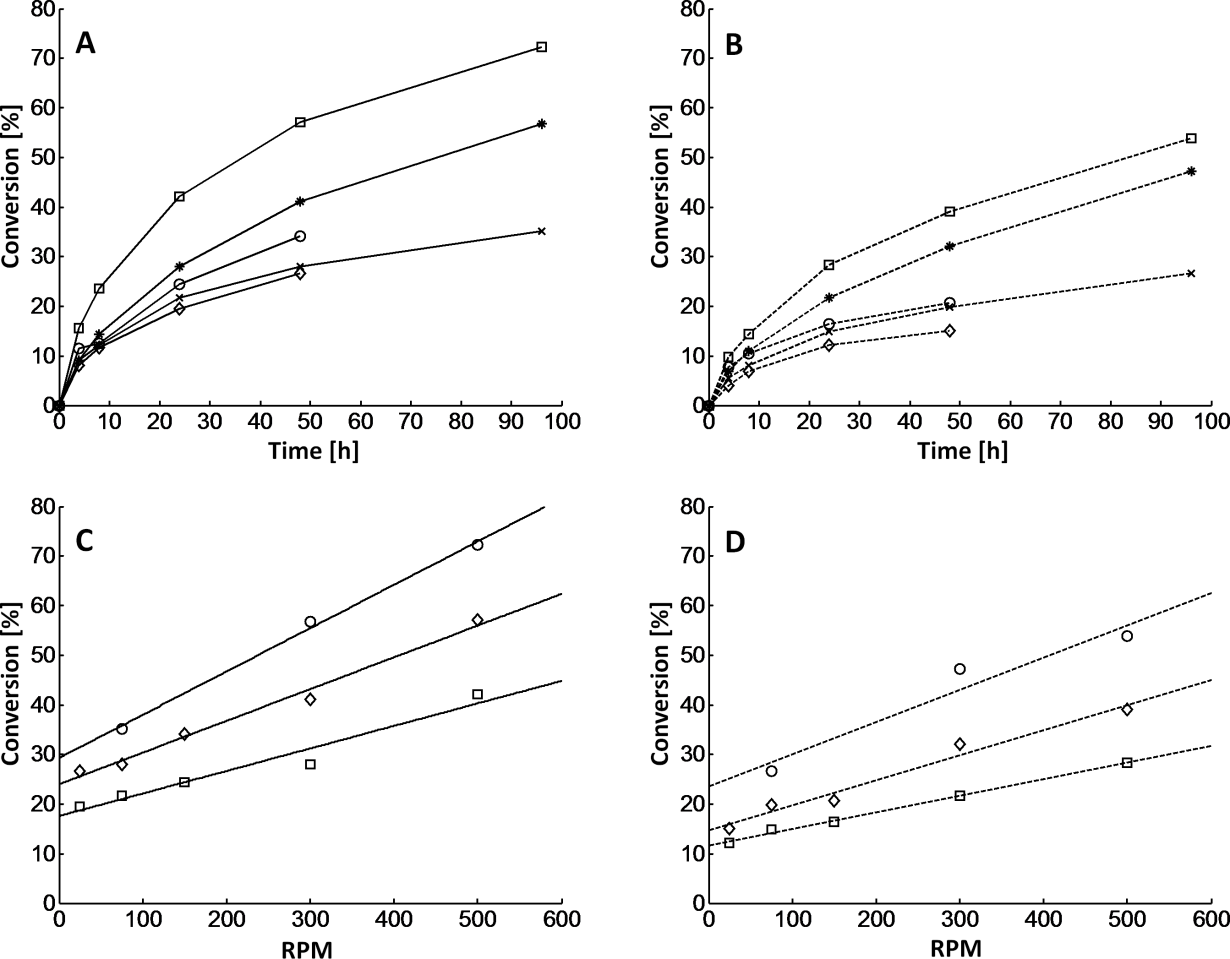
**Conversion during hydrolysis of pretreated spruce for different impeller speeds**. **(A, B) **Conversion during hydrolysis of pretreated spruce (10% water-insoluble solids; WIS) for different impeller speeds (squares = 500 rpm; stars = 300 rpm; circles = 150 rpm; crosses = 75 rpm, diamonds = 25 rpm). **(C, D) **Conversion at different impeller speeds for three selected times (Circles = 96 hours, diamonds = 48 hours, squares = 24 hours). **(A, C) **Solid lines = enzyme load of 20 FPU/g glucan; **(B, D) **dashed lines = 10 FPU/g glucan. The SD in the measured concentrations was < 4.2% for the duplicates.

The conversion of glucan to glucose was strongly affected by impeller speed (Figure 1a-b), for both high and low enzyme loadings (20 FPU/g glucan and 10 FPU/g glucan, respectively). At the highest impeller speed (500 rpm), the conversion after 48 h was more than twice as high as that seen with the lowest impeller speed (25 rpm), that is, 57% compared with only 26% conversion. Furthermore, an almost linear relationship between impeller speed and conversion was found for both enzyme loadings, and interestingly, the positive effect of mixing seemed to be maintained throughout the hydrolysis and did not decrease with time (Figure 1c-d). In fact, it seemed that the influence of mixing increased the further the hydrolysis proceeded, as indicated by the steeper slope of the curves.

Interestingly, as Metzner and Otto [21] have suggested, the average shear rate is proportional to the impeller speed in a stirred reactor, which indicates that the effects seen in these experiments could be related to shear forces exerted by the increased speed of the impeller.

### Estimation of power consumption

**Figure 2:**
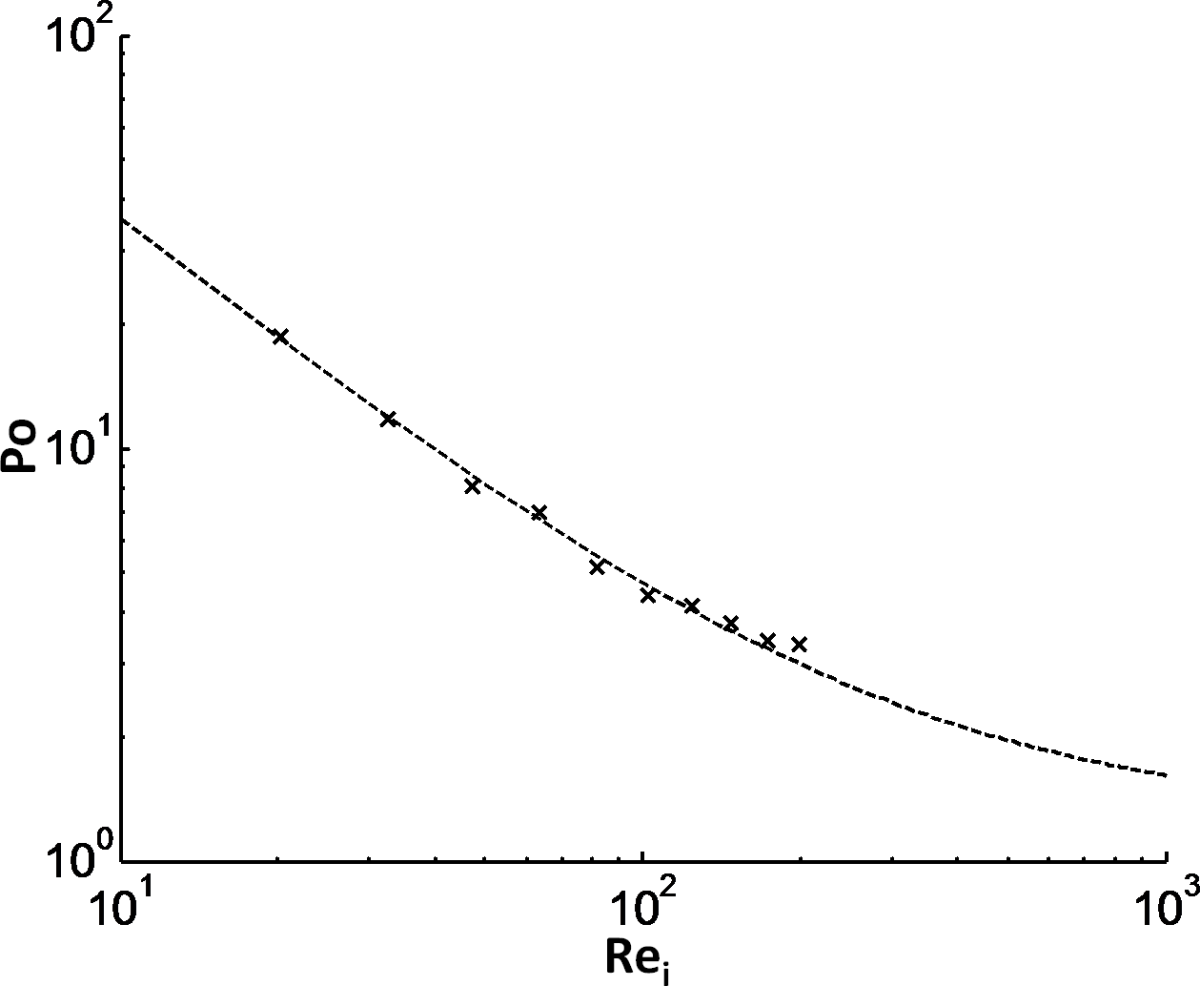
**Relationship between power number (P_o_) and Reynolds impeller number (Re_i_) for the reactor set-up**. Crosses indicate the calculated values based on the power measurements, and the dashed line represents the least-square fit to the equation, published previously [22].

**Figure 3:**
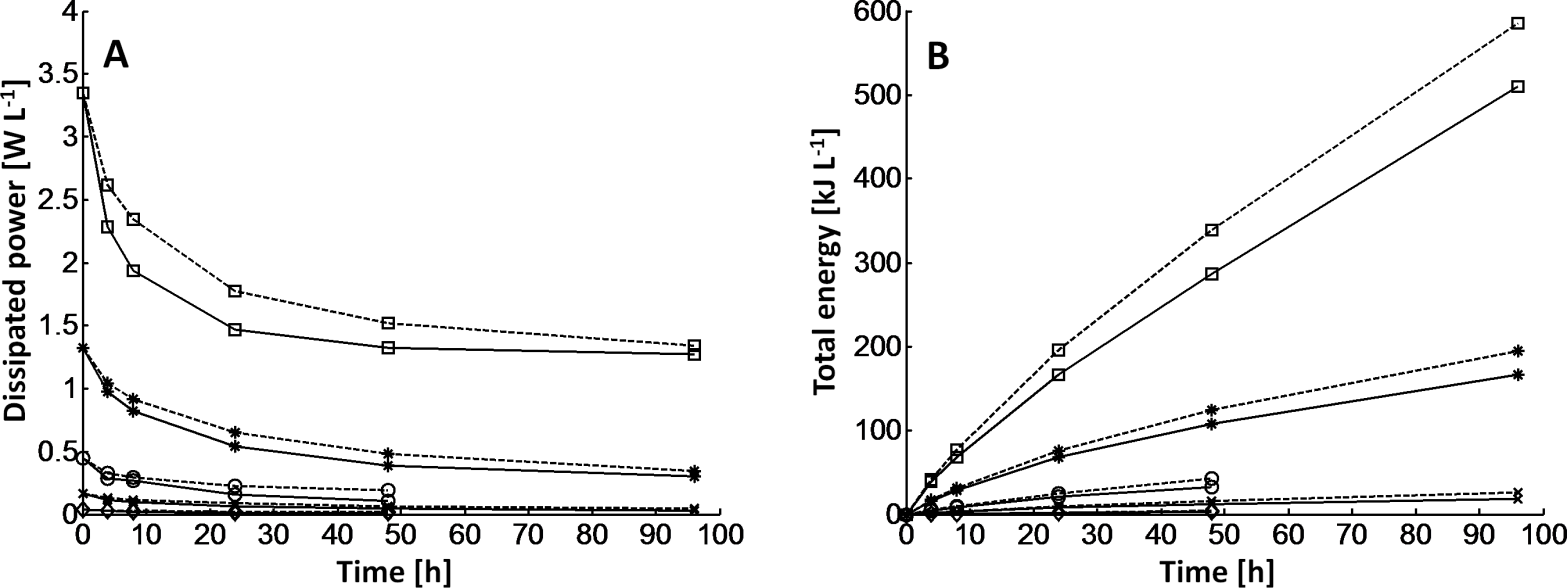
**Calculated dissipated power and total energy input during the hydrolysis of pretreated spruce for different impeller speeds**. **(A) **Calculated dissipated power and **(B) **total energy input during the hydrolysis of pretreated spruce (10% initial WIS content) for different impeller speeds (squares = 500 rpm, stars = 300 rpm, circels = 150 rpm, crosses = 75 rpm, diamonds = 25 rpm). Solid lines = enzyme load of 20 FPU/g glucan; dashed lines = 10 FPU/g glucan.

**Figure 4:**
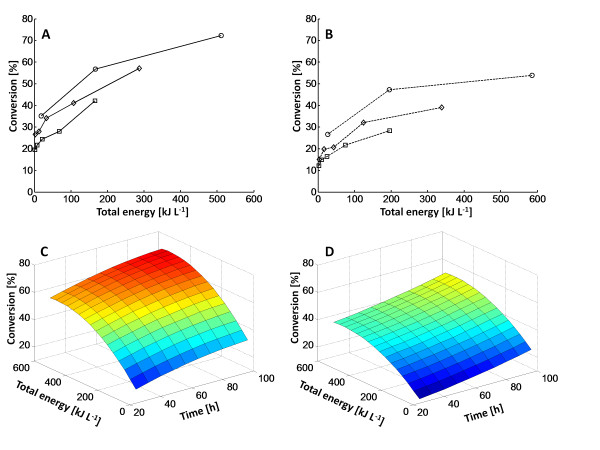
**Conversions obtained for enzymatic hydrolysis of pretreated spruce at corresponding energy input for three selected times and response surface area and hydrolysis time for the two different enzyme loadings**. **(A, B) **Conversions obtained for enzymatic hydrolysis of pretreated spruce at corresponding energy input for three selected times (circles = 96 hours, diamonds = 48 hours, squares = 24 hours). **(A) **Solid lines = enzyme load of 20 FPU/g glucan; **(B) **dashed lines = 10 FPU/g glucan. **(C**, **D) **Response surface area, where conversion is a function of total energy input and hydrolysis time for the two different enzyme loadings, **(C) **20 FPU/g glucan and **(D) **10 FPU/g glucan.

A relationship between power number (P_0_) and Reynolds impeller number (Re_i_) was established from the power measurements and the rheological data provided by Wiman *et al. *[2] (Figure 2). The data were fitted to the equation previously suggested by Wassmer and Hungenberg [22] (P_0 _= K_1_/Re_i _+ K_2_) to relate the power number to the Reynolds impeller number over a wide range of Re_i_. The fitted values for the parameters, K1 and K2, were 346.7 and 1.27 respectively. As the hydrolysis proceeded, the viscosity of the material decreased, which led to reduced power consumption (Figure 3a); that is, the viscosity reduction led to an increase in Reynolds impeller number, which in turn led to a decrease in power number (Figure 2). Clearly, the total dissipated energy during the hydrolysis increased drastically with increased impeller speed (Figure 3b). By increasing the enzyme dosage, the energy consumption could be reduced, because of faster hydrolysis of the material, which lowered the viscosity. It was therefore possible to reach similar conversions at different mixing energy input by altering residence time, impeller speed and/or enzyme load (Figure 4). In addition, it was clear that substantial amounts of energy were required to achieve only minor increases in conversion during the later stages of hydrolysis. A surface-response statistical model was used to illustrate the influence of energy input, residence time and enzyme loading on the conversion (Figure 4 c-d). First- and second-order terms were sufficient to describe the data satisfactorily (R^2 ^= 0.991 and 0.980 respectively for the different enzyme loads).

## Discussion

To date, efforts to improve the EH of pretreated lignocelluloses have mainly focused on the development of new pretreatment methods and new enzymes or enzyme mixtures. However, when scaling up the process, other factors need to be accounted for, and one of these is mixing, which affects both the efficiency of EH and the required energy input. In the current study, we examined the individual effects of and the interplay between mixing, energy input and enzyme loading. There was a marked difference in conversion, that is, rate of hydrolysis, for impeller speeds ranging between 25 and 500 rpm (Figure 1), and this effect remained important throughout the 96 hour reaction.

The increased hydrolysis rate seen at increased impeller speeds might be explained by the increased shear forces applied (because the average shear rate in the reactor is proportional to the impeller speed [21]). This may produce more rapid breakdown of the fibers, which in turn would increase accessibility for the enzymes. However, a mechanical 'pre-shearing' of the material before starting the hydrolysis had no significant effect on the hydrolysis in this study (results not shown), but it is still possible that once the hydrolysis has started, the fibers get 'weaker' and more sensitive to mechanical shearing. This phenomenon could be seen as a type of milling or even 'peeling' of the fibers, where oligomers of glucan are ripped from the larger fibers once they get partially hydrolyzed, making the underlying surface accessible to new enzymes. This explanation would be in line with the effect of mechanical shearing reported by Mais *et al. *[23]. Lenting and Warmoeskerken also suggested that mechanical shear forces could damage the cellulose fibers (mainly the crystalline regions) which would result in more amorphous regions (that is, better reaction sites) [24]. This phenomenon would be of greater importance the longer the hydrolysis proceeds, and may explain why the difference between conversions at different impeller speeds does not level out with time.

Mechanical breakage of the fibers, which generates new reactive surface areas, could enhance the adsorption of the enzymes. Better adsorption of enzymes when applying higher shear forces (for example from an increased impeller speed) have previously been reported [7,25,26]. Eriksson *et al. *also found, when measuring enzyme adsorption onto thin layers of cellulose, that the rate of both enzyme adsorption and hydrolysis increased with increasing impeller speed, a phenomenon they related to the shorter diffusion distance for the enzymes to the fibers [27]. A shorter diffusion distance is a result of a decrease in the film thickness of stagnant liquid surrounding each fiber, when increasing the impeller speed. Furthermore, a shorter diffusion distance for the hydrolysis products could potentially decrease local product inhibition because it would result in lower concentration of glucose/cellobiose close to the fibers (that is, the reaction site of endo- and exoglucanases). Local product inhibition would probably be more pronounced at low impeller speeds, and because the conversion increases almost linearly with impeller speed, product inhibition seems not to be the main effect.

Jorgensen *et al. *and Roche *et al. *suggested that achieving good initial distribution of the enzymes was the most crucial step and the degree of mixing subsequently was of less importance for the hydrolysis [5,6]. However, these studies used a rotating drum/barrel. Similar conclusions have been reported from studies using shake flasks [3,4]. In contrast to the agitated tank used in this work, a rotating drum is operated at low rotation speeds and relies on the mixing obtained when material falls to the bottom of the drum. The shear forces exerted in this system are likely to be significantly lower, and to be relatively independent of the rotation speed, which may explain why the rotation speed only had a minor influence. The findings from shake flask experiments could be similarly explained, because the exerted shear forces in this system are much lower than those in a stirred tank. However, it is, not possible from our experiments to fully determine what effects cause the increased hydrolysis rate. The results indicate an effect of higher shear forces, but further investigations, for example with different impeller types/sizes, are needed to verify this.

When comparing the estimated power consumptions in this study with values previously used in technoeconomical calculations [14] (about 60 W/m^3^) it was clear that much higher power inputs were needed in this work to achieve reasonable conversions. For example, the maximum conversion obtained in our experiments (72% after 96 hours) corresponded to a mean power requirement of about 1.5 kW/m^3^. In our experiments, an impeller speed of between 25 and 75 rpm would correspond to a mean energy consumption of about 50 W/m^3^; however, discouragingly low conversions were obtained for these stirrer rates. A strict comparison between power consumptions at largely different scales is difficult because of wall effects, as the area/volume of the reactor decreases with size. Another factor is that in small-scale, the reactor will operate in the laminar to transition region during most of the process, which means that the impeller power number will be relatively high (Figure 2). By contrast, at large-scale, the impeller diameter is much larger and it is likely that the reactor will operate in the turbulent region.

One way to reduce the power input for mixing was to add more enzymes to the reactor (Figure 3). This reduces the viscosity and, hence, the power consumption more rapidly. Doubling the amount of enzymes reduced the energy demand by 15 to 25%, depending on the impeller speed. In addition, increasing the amount of enzyme resulted in higher conversion (72% after 96 h at 20 FPU/g glucan, compared with only 54% at 10 FPU/g glucan at 500 rpm) (Figure 4). Similar glucan conversions could thus be achieved at different process conditions by altering enzyme load, energy input (that is, impeller speed) and/or residence time as indicated in Figure 4. The final choice of process parameters will of course be determined by the overall process economy.

An interesting process option, which has recently received attention, is the introduction of a liquefaction step [5] at high solid loadings, which precedes the SSF. Our results indicate that, even if this process configuration is used, it is still important to consider the effect of impeller speed in a hybrid SSF process. The reason is that the effect of mixing was shown to continue, and in fact even slightly increase, throughout the hydrolysis (Figure 1c-d).

## Conclusions

In conclusion, our results showed that the impeller speed had a large effect on the enzymatic conversion of pretreated spruce in a stirred tank reactor. The effect seemed to be proportional to the average shear rate in the reactor, implying that shear effects, such as mechanical milling or peeling of the fibers or shorter diffusion distance of the enzymes, could play an important role in the EH, even after 96 hours.

Additionally, these results indicate that higher power input for mixing, compared with those reported in previous technoeconomical evaluations, may be needed to obtain efficient EH.

## Competing interests

The authors declare that they have no competing interests.

## Authors' contributions

BP participated in the design of the study, performed the experimental work and wrote the manuscript. MW participated in the design of the study, performed the experimental work and commented on the manuscript. GL participated in the design of the study and commented on the manuscript. All authors contributed to the scientific discussion throughout the work, and have read and approved the final manuscript.
